# Analysis of the dynamics of transition from non-colonization to colonization and *Staphylococcus aureus* bacteremia in hemodialysis patients using Markov models.

**DOI:** 10.12688/f1000research.151896.2

**Published:** 2024-11-04

**Authors:** Daniela Montoya-Urrego, Johanna M Vanegas, J Natalia Jiménez, Difariney González-Gómez

**Affiliations:** 1 Grupo de investigación en Microbiología Básica y aplicada (MICROBA), Escuela de Microbiología, Universidad de Antioquia, Medellín, Antioquia, Colombia; 2 Escuela de Ciencias de la Salud, Universidad Pontificia Bolivariana, Medellín, Antioquia, Colombia; 3 Grupo de investigación Demografía y Salud, Facultad Nacional de Salud Pública, Universidad de Antioquia, Medellín, Antioquia, Colombia

**Keywords:** Markov models, Multistate models, Staphylococcus aureus, Hemodialysis, Colonization, Bacteremia.

## Abstract

**Background:**

Hemodialysis patients are frequently colonized by *Staphylococcus aureus*, leading to severe infections with high mortality rates. However, little is known about transition from non-colonization to colonization or bacteremia over time. The aim was to analyze the behavior of *S. aureus* colonization, identifying the probability of transition from non-colonized to colonized state or bacteremia, and the influence of specific covariates.

**Methods:**

The study was conducted in a dialysis unit associated with a tertiary care hospital in Medellín between October 2017 and October 2019. An initial measurement was taken to evaluate *S. aureus* colonization, and follow-up measurements were performed 2 and 6 months later. Bacteremia evolution was monitored for 12 months. A two-state recurrent continuous-time Markov model was constructed to model transition dynamics from non-colonization to *S. aureus* colonization in hemodialysis patients. Subsequently, the model was applied to a third state of bacteremia.

**Results:**

Of 178 patients on hemodialysis, 30.3% were colonized by *S. aureus.* Transition intensity from non-colonization to colonization was three times higher (0.21; CI: 0.14-0.29) than from colonization to non-colonization (0.07; CI: 0.05-0.11). The colonization risk increased in patients with previous infections (HR: 2.28; CI: 0.78-6.68), hospitalization (HR: 1.29; CI: 0.56-2.99) and antibiotics consumption (HR: 1.17; CI: 0.53-2.58). Mean non-colonized state duration was 10.9 months, while in the colonized state was 5.2 months. In the 3-state model, it was found that patients colonized were more likely to develop *S. aureus* infection (13.9%).

**Conclusion:**

A more likely transition from non-colonization to colonization was found, which increases with factors such as previous infection. In addition, the development of bacteremia was more likely in colonized than in non-colonized patients. These results underline the importance of surveillance and proper management of *S. aureus* colonization to prevent serious complications, such as bacteremia, and improve prognosis in this vulnerable population.

## Introduction

*Staphylococcus aureus* is one of the microorganisms that most frequently colonizes and causes invasive infection, such as bacteremia in hemodialysis patients. ^1^ In addition to increasing the risk of endogenous infection by up to 77.8% in this group of patients, colonization by *S. aureus* favors the dissemination of this bacterium at hospital and community level, due to the fact that colonized patients constantly circulate among these environments and act as asymptomatic reservoirs and carriers of the microorganism for long periods of time. ^2^^,^^3^ The situation become more worrying when isolates are methicillin-resistant (MRSA), because treatment options are reduced, the prognosis for the patient worsens and morbidity, mortality and the cost of care increase significantly. ^1^

Colonization by *S. aureus* in hemodialysis patients can be persistent, which refers to the permanent presence of the microorganism over time; or intermittent, in which the microorganism can be present or absent at different times. ^3^^,^^4^ There is little information on the probability of switching between the non-colonized state and the colonized state and vice versa, as well as the period of permanence of patients in each state and the implications for infection. This is important because, if patients remain colonized for a longer period of time, this favors the risk of invasive infections such as bacteremia, since the colonizing bacteria can contaminate catheters or other devices and reach the bloodstream. ^5^

In this sense, Markov models emerge as a valuable and useful statistical tool to study the dynamics of colonization over time, allowing to estimate the transition between states, in this case from a state of non-colonization to one of colonization or infection. ^6^^,^^7^ This type of statistical model allows modelling systems that change randomly, and assumes that future states depend only on the current state, not on the events that occurred before it (a property known as the Markovian assumption). ^6^

By focusing on this property, Markov models enable estimation not only of the probability of being in each state at any given time but also of the expected duration in each state before transitioning. This ability is essential for predicting patterns over time, as it enables modeling of state behaviors calculation of transition rates, and the stability or persistence within each state. Thus, Markov models provide a structured approach to understand and predict patient transition between non-colonization, colonization, and infection states, offering important insights into the temporal dynamics of colonization and its clinical implication.

In this study, we proposed to analyze the behavior of colonization by *S. aureus*, identifying the probabilities of change from non-colonized to colonized states and bacteremia, and the influence of some covariables in these transitions. This will allow designing and directing strategies to avoid progression to states that compromise the patient’s prognosis, such as colonization and infection.

## Methods

### Study population

A cohort of 210 hemodialysis patients was taken from a previous study that was carried out in a dialysis unit in Medellín, Colombia, in which colonization by *S. aureus* was evaluated at three moments in time: at the beginning of the study, at two and at 6 months; and the development of bacteremia during a 12-month follow-up. ^2^ This study included patients from a dialysis unit associated to a high complexity hospital in Medellín, over 18 years of age, with chronic kidney disease and central venous catheter on hemodialysis. Patients who had only the baseline measurement were excluded from the present study. Informed consent was signed by each patient.

### Variables

Colonization was assessed in nostrils and skin around the insertion of the hemodialysis catheter at the beginning of the study, at 2 and 6 months later, in order to capture and identify all potential transitions and the behavior of colonization. ^8^^–^^10^ Infection was defined as the diagnosis of bacteremia according to the criteria given by the Center for Disease Control and Prevention (CDC), as the presence of fever, chills or hypotension with bacteria identified in the blood and not related to an infection at any other site. ^11^ Central-line associated bloodstream infections (CLABSI) were also considered in the analysis.

Additionally, demographic and clinical variables were evaluated, such as age, sex, smoking, history of hospitalization, previous infection, antibiotic use, comorbidities, and catheter or fistula use. To apply the Markov model, two possible recurrent states were initially considered: 1: Non-colonized and 2: Colonized. Subsequently, at a second stage, 3 states were considered: 1: Non-colonized, 2: Colonized and 3: Bacteremia. Consequently, individuals can independently transit between states where the probability of transition is not time-dependent. ^12^ Transitions are defined as changes from one state to another, and this process can be specified in terms of transition intensities. For each patient a transition history was established based on a maximum of three observations during the follow-up period, in which the state of colonization, non-colonization or infection was determined. It is important to highlight that in the renal unit where the study was conducted, there were no decolonization protocols in place for patients. As a result, this factor did not influence the transitions between states.

### Data collection

The clinical and epidemiological information was obtained by a questionnaire designed for this purpose, which was applied to all the hemodialysis patients in the company of a researcher by interview. Similarly, the clinical histories provided by the dialysis center were taken into account to know the clinical history of patients.

### Colonization screening

To detect *S. aureus* colonization, samples were obtained from nostrils and skin around the catheter insertion, using a sterilized cotton swab with sterile 0.9% saline solution. Each swab was transported in AMIES medium (transport medium with activated carbon) and then enriched for 18 to 24 hours at 37°C in trypticase soy broth (TSB- OXOID ^TM^, CM0129), prepared according to manufacturer’s instructions, adding 30 g to 1 liter of water. Subsequently, it was plated on mannitol salt agar for the selection of fermenting colonies indicative of *S. aureus.* Preliminary identification was performed by phenotypic methods based on colony morphology in sheep blood agar and positive catalase and coagulase tests. Identification of isolates and antibiotic susceptibility was determined using the Vitek ^®^-2 automated system (bioMérieux) according to Clinical and Laboratory Standards Institute (CLSI) cut-off points. ^13^

### Statistical analysis

Categorical variables were described as absolute and relative frequencies. Quantitative variables were expressed as mean and standard deviation or median and interquartile range, according to the assumption of normality. Subsequently, Markov models were applied, in which the probability of transitioning from one state to another depends solely on the current state, with no influence from past states, satisfying the Markov property. ^6^ To estimate the transition rates and probabilities between states, the inter-occurrence times were transformed such that the time of the first measurement was considered as time zero (t0), and subsequent measurements were considered as time 1 (t1) and time 2 (t2). This approach allows estimating the transition intensity functions between states, identifying all the transitions that occurred between observations, estimating the maximum probability of transition from one state to another and estimating the maximum probabilities when covariables are present. ^6^^,^^14^

In relation to the above, two transition matrices were calculated: the transition count matrix *Q* and the transition probability matrix *P.* The transition matrix *Q* represents the frequency of individuals transitioning from state *r* to state *s* during a given time interval, called by *q _rs_*. Each element of this matrix counts the number of transitions observed between specific states during the follow-up period. The transition probability matrix *P,* that refers to the probability of moving from state *r* to state *s* in the time interval *p _rs_,* is calculated by dividing each of the elements of matrix *Q* by the row total. ^15^

In both matrices, the rows are designate by the current state, and the columns represent the state to which the transition may occur. The sum of the probabilities of a row of the transition matrix is equal to 1. The transition probabilities are presented with the 95% confidence interval. For the case of patients with *S. aureus* all possible transitions between states are allowed, in all states there is a positive probability of reaching it.

Statistical analyses were conducted using R software, version 4.4.1, specifically employing the msm Markovchain package. Additionally, the analysis code can be found in the GitHub repository (Extended data).

## Results

Of 210 hemodialysis patients, 32 were excluded from the present study because only had the initial observation, leaving a total of 178 patients included. Of the excluded patients, 25% (n=8) passed away, while the rest had only one measurement for other reasons, such as transferring to another renal unit, voluntarily withdrawing from the study, receiving a kidney transplant, or discontinuing treatment by choice (see extended data). The majority of patients were female (51.7%, n=92), and the mean age was 62 years (SD 15.9). Clinical data revealed that 68.5% (n=122) of patients had been hospitalized in the last year, while 57.3% (n=102) consumed antibiotics in the previous 6 months. The most common comorbidities were diabetes mellitus (44.4%, n=79), heart failure (24.7%, n=44), and coronary artery disease (20.2%, n=36). At baseline, 30.3% (n=54) of patients had *S. aureus* colonization.

### Two recurrent state models for *S. aureus* colonization

**Figure 1.**
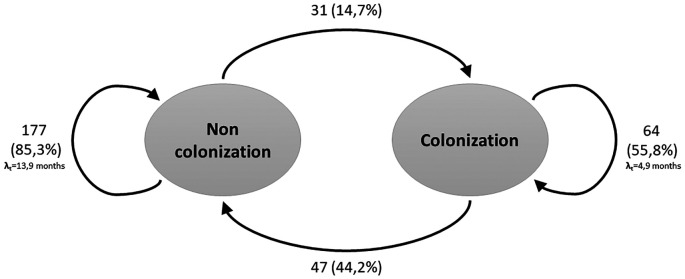
Transitions between two recurrent states of the Markov model for hemodialysis patients. The arrows indicate the allowed transitions between states. Patients can remain in one state in consecutive cycles.

The recurrent two-state model (Non-Colonized - Colonized) is shown in Figure 1. During the follow-up period, and considering all three measurements, the transition intensity was 3 times higher for going from a non-colonized to colonized state (0.21; CI: 0.14-0.29) compared to the transition from colonized to non-colonized state (0.07; CI: 0.05-0.11). The probabilities of remaining in the same state decreased over time, contrary to the transition between the two states, which presented higher likelihoods over time ( Table 1).

**Table 1.** Transition probability and intensity in the two-state Markov model.

Transition	Transition intensity (IC 95%)	Transition probability		
		Baseline	2 months	6 months
Non-colonized → Non-colonized	-0.07 (-0.11; -0.05)	0.93	0.89	0.79
Non-colonized → Colonized	0.21 (0.14; 0.29)	0.07	0.11	0.21
Colonized → Non-colonized	0.07 (0.05; 0.11)	0.18	0.32	0.60
Colonized → Colonized	-0.21 (-0.29; 0.14)	0.82	0.68	0.40

### Effect of covariates on the dynamics of two-state colonization by *S. aureus*

In the presence of most of the covariates analyzed, such as smoking, previous hospitalization, antibiotic use and comorbidities, the transition intensities from the colonized to the non-colonized state doubled with respect to the transition intensities from the non-colonized to the colonized state, with the exception of previous infection, which presented similar intensities in both transitions ( Table 2).

**Table 2.** Transition intensity and risk ratios for covariates in recurrent two-state model in *S. aureus* colonization.

Variable	Hazard Ratio of transition		Transition intensity			
	Non-colonized → Colonized (IC 95%)	Colonized → Non-colonized (IC 95%)	Non-colonized → Non-colonized (IC 95%)	Non-colonized → Colonized (IC 95%)	Colonized → Non-colonized (IC 95%)	Colonized → Colonized (IC 95%)
**Smoking**	0.67 (0.29;1.53)	1.62 (0.78;3.33)	-0.07 (-0.11;-0.04)	0.07 (0.04; 0.11)	0.21 (0.15;0.30)	-0.21 (-0.30;-0.15)
**Hospitalization**	1.29 (0.56;2.99)	1.62 (0.76;3.46)	-0.070(-0.11;-0.05)	0.07 (0.05;0.11)	0.21 (0.15;0.30)	-0.21(-0.30;-0.14)
**Previous infection**	2.28 (0.78;6.68)	1.22 (0.41;3.63)	-0.18 (-0.45;-0.07)	0.18 (0.07;0.45)	0.19 (0.07;0.50)	-0.19 (-0.50;-0.07)
**Antibiotics consumption**	1.17 (0.53;2.58)	0.97 (0.47;1.99)	-0.07 (-0.11;-0.05)	0.07 (0.05;0.11)	0.20 (0.14;0.29)	-0.20 (-0.29;-0.14)
**Diabetes mellitus**	0.58 (0.26;1.33)	1.08 (0.53;2.18)	-0.07 (-0.10;-0.05)	0.07 (0.05;0.10)	0.21 (0.14;0.29)	-0.21 (-0.29;-0.15)
**Heart failure**	2.0 (0.84;4.78)	1.65 0.72;3.76)	-0.15 (-0.31;-0.07)	0.15 (0.07;0.31)	0.27 (0.13;0.55)	-0.27 (-0.55;-0.13)
**Coronary artery disease**	1.27 (0.50;3.18)	1.33 (0.52;3.38)	-0.11 (-0.24;-0.05)	0.11(0.05;0.24)	0.23 (0.09;0.55)	-0.23 (-0.55;-0.10)
**Arterial hypertension**	0.21 (0.05;0.84)	0.76 (0.16;3.70)	-0.07 (-0.11;-0.05)	0.07 (0.05;0.11)	0.21(0.15;0.31)	-0.21 (-0.31;-0.15)
**Charlson index**	1.01 (0.86;1.18)	1.10 (0.96;1.26)	-0.09 (-0.13;-0.06)	0.09 (0.06;0.13)	0.19 (0.14;0.27)	-0.19 (-0.27;-0.14)
**Karnofsky index**	0.99 (0.97;1.03)	0.98 (0.95;1.01)	0-.09 (-0.13;-0.06)	0.09 (0.06;0.13)	0.18 (0.13;0.26)	-0.18 (-0.26;-0.13)

On the other hand, no evidence was found of the influence of the covariates studied on transition risk. However, significant tendencies to exceed the risk threshold for transition from the non-colonized state to the colonized state were observed in the presence of previous infection (HR: 2.28; CI: 0.78-6.68), previous hospitalization (HR: 1.29; CI 0.56-2.99), antibiotic consumption (HR: 1.17; CI 0.53-2.58) and heart failure (HR: 2.00; CI 0.84-4.78). On the other hand, the factors that showed a tendency to increase the transition risk from colonized to non-colonized state were history of hospitalization (HR: 1.62, CI 0.76-3.46) and smoking (HR:1.62; CI: 0.78-3.33). However, these risk indices were not significant ( Table 2).

Regarding the average time spent (in months) in one of the two states analyzed, it was found that there was longer duration of colonization in presence of hypertension (5.33; CI: 3.72-7.63) and in patients with previous antibiotic use (4.98; CI: 2.86-8.65).

### Three-state model for *S. aureus* colonization

In a second stage of the analysis, a third condition was incorporated: *S. aureus* bacteremia. Regarding this, 35.2% (n=64) of the patients were found to be in the colonized state, 64.8% (n=118) in the Non-colonized state and 4.39% (n=23) in the infected state.

**Figure 2.**
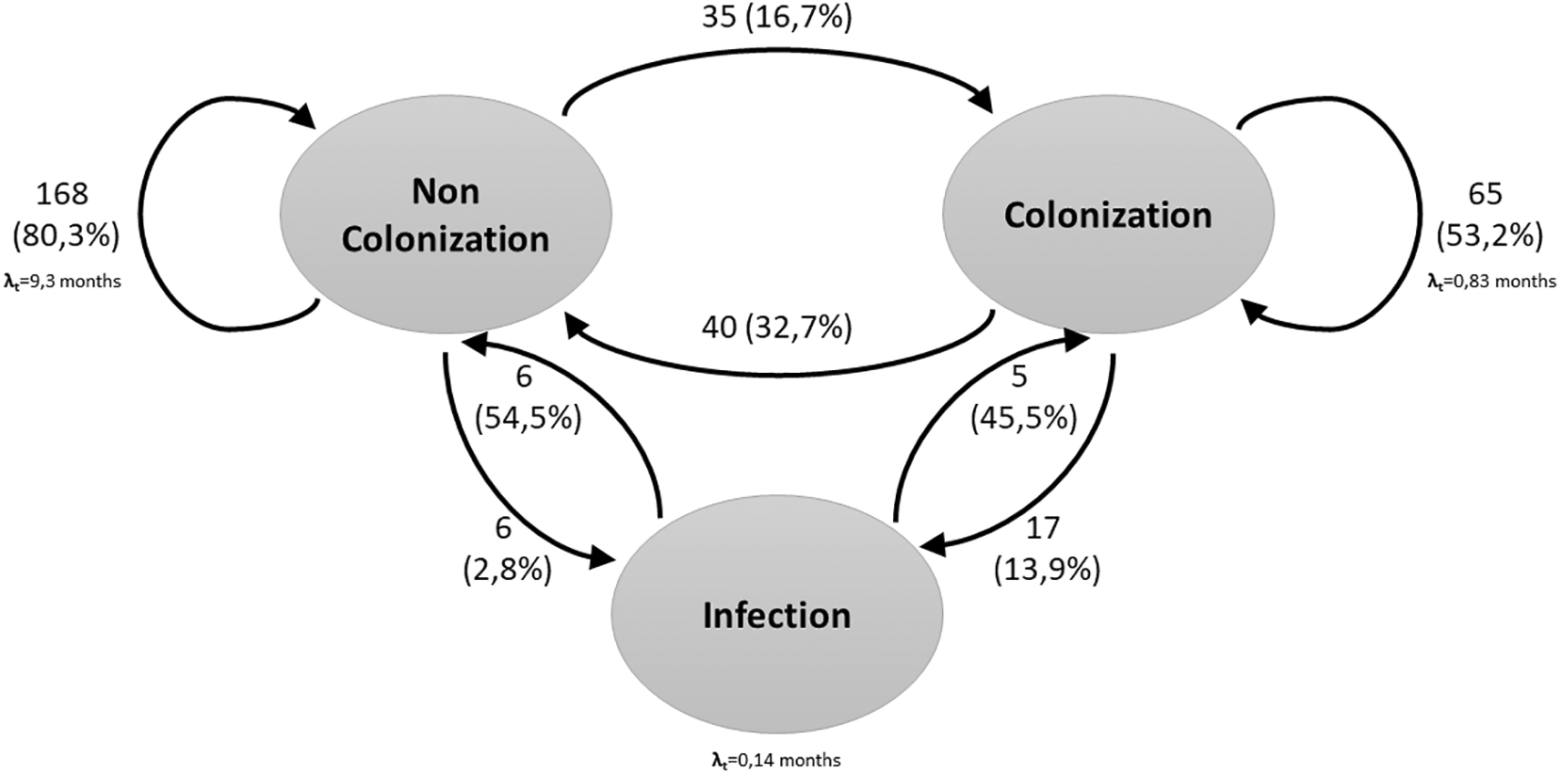
Three-state Markov model for *S. aureus* colonization and infection. The arrows indicate the allowed transitions between states. Patients can remain in one state in consecutive cycles.

In the three-state model, the results were similar to those presented in the two-state model. There was a 16.7% probability of moving from the non-colonized state to the colonized state. The probability of moving from the colonized state to the non-Colonized state was 32.7%. Staying in the non-Colonized state had a probability of 80.3%, while in the colonized state had a probability of 53.2%. On the other hand, the probability of a non-colonized person becoming infected was 2.8%, and among the colonized patients, 13.9%. There was also a longer stay in the Non-Colonized state (9.3 months) compared to the Colonized state (25 days) and the Infected state (4 days) ( Figure 2).

Related with the probability of moving from the Non-Colonized to Colonized state, it increased from the baseline measurement to the 12-month measurement (from 0.11 to 0.27), as did the probability of moving from Non-Colonized to Infected (from 0.03 to 0.06). In contrast, the probability of moving from colonized to infected status decreased over time, from 0.11 at baseline to 0.06 at 12 months ( Table 3).

**Table 3.** Estimation for the probability of transition between the 3-state model.

Transition	Transition probability		
	2 months	6 months	12 months
Non-colonized → Non-colonized	0.85	0.72	0.67
Non-colonized → Colonized	0.11	0.23	0.27
Non-colonized → Infected	0.03	0.05	0.06
Colonized → Non-colonized	0.26	0.53	0.63
Colonized → Colonized	0.62	0.40	0.31
Colonized → Infected	0.11	0.08	0.06
Infected → Non-colonized	0.35	0.56	0.64
Infected → Colonized	0.55	0.37	0.30
Infected → Infected	0.10	0.07	0.06

## Discussion

In this study, Markov models were used to analyze the behavior of *S. aureus* in hemodialysis patients, and to predict its change between the states of non-colonization, colonization and bacteremia over time. Transition models have positioned as a powerful tool for studying transitions from one state to another, allowing to shed light on behaviors and outcomes of interest in some diseases and infections, such as smoking, diabetes and cancer. ^16^^–^^18^ Unlike other estimation models, such as Generalized Estimating Equations (GEE), with Markov models it is possible to simultaneously study transitions in both directions, such as from non-colonized to colonized and from colonized to non-colonized. In addition, make it possible to identify factors that may behave differently from one direction to the other. ^19^ Particularly in the case of the use of Markovian models to evaluate *S. aureus* colonization, several studies have been reported. ^20^^–^^22^ However, the application of Markov models to evaluate the transition of states in hemodialysis patients presents limited evidence.

Considering the above, the results of this study show that, for patients who started in a non-colonized state, the probability of changing to a colonized state was 14.7%, lower than the probability of changing from the colonized to the non-colonized state ( Figure 1). However, the transition intensity was found to be 3 times higher in the switch from the non-colonized to colonized state ( Table 1). This may be due to the fact that this type of patient has specific baseline conditions and clinical characteristics that favor colonization, such as high antibiotic consumption, presence of different comorbidities, constant transit between the community and medical centers, frequent hospitalizations and regular contact with medical personnel and other patients. ^8^^,^^23^ Similarly, in the few studies that have used Markovian models to understand the behavior of *S. aureus* in other populations, they reach the same conclusion, demonstrating that previous antibiotic use is related to the acquisition of Methicillin-Resistant *S. aureus* (MRSA) strains in nursing homes. ^22^^,^^24^ This similarity may be due to the fact that, in this type of population, as well as in hemodialysis patients, there is an environment with conditions that favor colonization by this type of bacteria.

On the other hand, trends were found that the risk of passing from the non-colonized state to the colonized state increased when the patient had a previous infection, previous hospitalization, and antibiotics consumption; also, that the time spent in the colonized state increased when the patient had arterial hypertension and previously used antibiotics. This is in agreement with what has been reported by other authors, who have found an association between previous hospitalization and the risk of *S. aureus* colonization, and reinforce the importance of the previous conditions of hemodialysis patients in colonization. ^25^^,^^26^ Likewise, it has been described that most invasive *S. aureus* infections are of endogenous origin in these patients, ^27^ therefore, remaining in a colonized state for a longer period of time increases the risk of developing an infection by this bacterium, such as bacteremia. ^4^ Carrier status increases the spread of the microorganism at the hospital and community level, since patients constantly circulate between these two environments, and have a care link, not only with health personnel but also with their home contacts and general community. ^8^^,^^27^^,^^28^

Regarding the 3-state model, in which infection was considered, it was found that there is a greater probability that a colonized person will develop an infection compared to a non-colonized person. This is in agreement with what has been described in previous studies, which show that colonized persons have a higher risk of infection, especially bacteremia ^5^; it has even been reported that 77.85% of hemodialysis patients who developed bacteremia due to *S. aureus* were previously colonized. ^2^ In addition, the need to prevent colonization that can lead to infection is emphasized, due to the great implications in morbidity, mortality and worse prognosis of patients. ^29^^,^^30^ On the other hand, it was found that the time spent in the non-colonization state is longer than the time spent in colonization or infection, this may be due to the fact that patients go from colonization to infection or that they receive antibiotic treatment. This may also explain why the probability of remaining in the same state decreases over time.

Finally, high frequencies of colonization and infection in hemodialysis patients demonstrate the importance of maintaining active epidemiological surveillance in order to take actions to prevent infection. One way is to evaluate the possibility of establishing decolonization protocols that reduce the incidence of infections. ^31^ In a study that also used Markov models to model the decolonization of *S. aureus*, it was shown that the use of mupirocin as a therapy to decolonize patients’ nostrils was effective and reduced autoinoculation and infection. ^21^ In the same sense, infections by this bacterium generate a high cost in the health system, especially in hemodialysis patients, so being able to predict the behavior of *S. aureus* from colonization to infection can reduce the economic burden, both for the health system and for the patient and their family. ^32^^,^^33^

Regarding limitations, this study was conducted at a single facility, which means it may not accurately represent the epidemiology in other dialysis centers. However, the institution where the research was conducted is one of the largest in the city and serves a diverse population from various areas. On the other hand, although *S. aureus* colonization can occur in multiple body sites, we only evaluated nasal colonization and the skin around the catheter, taking into account that the nasal region is the most common and is considered a strong indicator of overall colonization, offering high sensitivity for detecting *S. aureus* colonization. ^34^^,^^35^ Likewise, it was not possible to perform a separate analysis of the behavior of MRSA and MSSA due to the limited number of patients colonized by MRSA. Additionally, the inclusion of patients at an arbitrary point during hemodialysis treatment introduces variability in the interpretation of colonization transition intensities, especially with respect to how these may change over the time of hemodialysis therapy. Since *S. aureus* colonization may occur at different stages of treatment, the time from the start of hemodialysis to the time of inclusion in the study could influence the observed colonization patterns.

While the Markov model is really useful to study the transitions between non-colonization, colonization and infection by *S. aureus* in hemodialysis patients, it is important to clarify that this model does not take into account that this dynamic is also affected by the specific epidemiology of each institution and by the “Colonization pressure”, ^36^ which varies according to the number of colonized patients in the renal unit. This limitation restricts the generalizability of our study to other contexts. Similarly, factors such as hospital stays, antibiotic use, and use of medical devices can affect the transitions between states, making them not constant over time, as assumed in the Markov model, which may affect the clinical applicability of the model. Nonetheless, multiple studies have demonstrated that colonization significantly increases the risk of subsequent infection. ^37^^,^^38^ Additionally, for patients who only had the initial measurement, death or receiving a kidney transplant were not considered competing events. Therefore, different types of risk analysis are necessary to address this limitation in future studies. Finally, in stochastic models, the evolution of a system may differ, even when starting from the same initial conditions. This is aggravated by the impossibility of controlling all the factors that influence the study of the phenomenon, which leads us to resort to probabilistic parameters.

## Conclusion

The Markov model are a tool that can be used to determine the behavior between states of non-colonization, colonization, and *S. aureus* bacteremia in hemodialysis patients. The evidence of a more likely transition from non-colonization to colonization, especially favored by factors such as previous infections and antibiotic use, highlights the need to adequately manage *S. aureus* colonization and to have strategies to prevent it. Likewise, this study showed a higher probability of developing bacteremia in colonized patients, which draws attention to the importance of early intervention of colonization in order to avoid its progression. Finally, the use of modeling tools to address this type of problem allows the continuation and improvement of active epidemiological surveillance, making it possible to identify, design and implement evidence-based strategies aimed at preventing colonization and infection in these patients.

## Ethics and consent

This research project conforms to the international ethical standards set forth in the Nuremberg Code, the Declaration of Helsinki, the Belmont Report and the World Health Organization’s Good Clinical Practice recommendations. The research was approved by the Ethics Committee for Human Investigations of the University of Antioquia (CBEIH-SIU) with the approval act No 17-65-689 dated May 3, 2017. Informed consent was signed by each patient.

## Data Availability

*Ethical and security consideration* The study data have sensitive and private information about the patients who participated. If the reviewers or the reader need access to the data, they can request it by e-mail to the corresponding author ( difariney.gonzalez@udea.edu.co). The data can be sent as long as it does not conflict with the consent signed by the patients. Figshare: Clinical and epidemiological information collection form. https://doi.org/10.6084/m9.figshare.26170027. ^39^ Figshare: Markov Models. https://doi.org/10.6084/m9.figshare.27313296.v1. ^40^ Figshare: Follow-up Hemodialysis Patients. https://doi.org/10.6084/m9.figshare.27313290.v1. ^41^ Data are available under the terms of the Creative Commons Attribution 4.0 International license (CC-BY 4.0).
