# Supplementary material for: Analysis of the dynamics of transition from non-colonization to colonization and Staphylococcus aureus bacteremia in hemodialysis patients using Markov models
Source: F1000Res. 2025 Jan 6;13:837. Originally published 2024 Jul 26. [Version 3] doi: 10.12688/f1000research.151896.3 (PMC11809629; doi:10.12688/f1000research.151896.3)
Supplement: Peer review history

# Reviewer report: Reviewer response for version 3

**DOI:** 10.5256/f1000research.176351.r356533

**Published:** 2025-01-07

**Authors:** Stephan Harbarth, Nasreen Kheir

**Affiliations:** 1 Geneva University Hospitals, Geneva, Switzerland

Thanks a lot.

 We went through the revised MS version, and think we can now end this story, the authors responded to all comments.

 No more issues from our side,

 Best wishes,

 KHEIR HASSOUN Nasreen & 

 HARBARTH Stephan

Is the work clearly and accurately presented and does it cite the current literature?

Partly

If applicable, is the statistical analysis and its interpretation appropriate?

Yes

Are all the source data underlying the results available to ensure full reproducibility?

Yes

Is the study design appropriate and is the work technically sound?

Yes

Are the conclusions drawn adequately supported by the results?

Yes

Are sufficient details of methods and analysis provided to allow replication by others?

No

Reviewer Expertise:

NA

We confirm that we have read this submission and believe that we have an appropriate level of expertise to confirm that it is of an acceptable scientific standard.

# Reviewer report: Reviewer response for version 2

**DOI:** 10.5256/f1000research.174101.r337447

**Published:** 2024-12-05

**Authors:** Thanaa Rasheed Abdulrahman

**Affiliations:** 1 Department of Microbiology, University of Al-Nahrain, Baghdad, Iraq

Greetings

 All is well and the author is committed to making corrections

 With my respects and best regards

Is the work clearly and accurately presented and does it cite the current literature?

Yes

If applicable, is the statistical analysis and its interpretation appropriate?

Yes

Are all the source data underlying the results available to ensure full reproducibility?

Partly

Is the study design appropriate and is the work technically sound?

Yes

Are the conclusions drawn adequately supported by the results?

Yes

Are sufficient details of methods and analysis provided to allow replication by others?

Partly

Reviewer Expertise:

NA

I confirm that I have read this submission and believe that I have an appropriate level of expertise to confirm that it is of an acceptable scientific standard.

# Reviewer report: Reviewer response for version 2

**DOI:** 10.5256/f1000research.174101.r337446

**Published:** 2024-11-18

**Authors:** Stephan Harbarth

**Affiliations:** 1 Geneva University Hospitals, Geneva, Switzerland

Thank you for the invitation to review the updated manuscript. The authors completed a thorough revision of their manuscript and addressed our previous concerns. They now explain the rationale for using Markov models for this analysis, making this manuscript clearer and more accessible for readers without a background in mathematical modelling. They included extended data and additional information needed for the reader while interpreting the study findings. Finally, they added a limitations section, summarizing all the points we raised in our review process. After reading the revised manuscript we have a few comments to be addressed:

 Major comment:

- The authors present hazards ratio estimates for transition between different states under “ *Effect of covariates on the dynamics of two-state colonization by S. aureus*” section, yet it is not clear what methods were used to obtain these estimates, and how does it fit within the modelling framework. This should be better explained in the statistical analysis in the methods section.

Minor comments:

- In the definition of bacteremia, it would be more accurate to state that primary bloodstream infection (BSI) and catheter related BSI (CLABSI) according to the CDC criteria were included. As in CLABSI a clear source of infection is identified (catheter) and in the current phrasing is contradictory: “ *Infection was defined as the diagnosis of bacteremia according to the criteria given by the Center for Disease Control and Prevention (CDC), as the presence of fever, chills or hypotension with bacteria identified in the blood and not related to an infection at any other site. Central-line associated bloodstream infections (CLABSI) were also considered in the analysis*”.
- The last sentence in the conclusions is not supported by the study findings: ” *Finally, the use of modeling tools to address this type of problem allows the continuation and improvement of active epidemiological surveillance, making it possible to identify, design and implement evidence-based strategies aimed at preventing colonization and infection in these patients*.” The authors could argue instead on the utility of modelling studies to evaluate decolonization or other infection control interventions in this patient population to inform future real-world epidemiological and interventional studies.

Is the work clearly and accurately presented and does it cite the current literature?

Partly

If applicable, is the statistical analysis and its interpretation appropriate?

Yes

Are all the source data underlying the results available to ensure full reproducibility?

Yes

Is the study design appropriate and is the work technically sound?

Yes

Are the conclusions drawn adequately supported by the results?

Yes

Are sufficient details of methods and analysis provided to allow replication by others?

No

Reviewer Expertise:

NA

I confirm that I have read this submission and believe that I have an appropriate level of expertise to confirm that it is of an acceptable scientific standard, however I have significant reservations, as outlined above.

# Response

**Published:** 2024-12-24

**Authors:** Daniela Montoya Urrego

**Affiliations:** Escuela de Microbiología, Universidad de Antioquia, Medellín, Antioquia, Colombia

Dear reviewers,

 Thank you for guidance reviewing our submission. The manuscript has been revised and your comments have been directly addressed. We believe that these revisions lead to a significantly improved manuscript and we hope it is now acceptable for publication.

**Major comment:**

*The authors present hazards ratio estimates for transition between different states under “Effect of covariates on the dynamics of two-state colonization by S. aureus” section, yet it is not clear what methods were used to obtain these estimates, and how does it fit within the modelling framework. This should be better explained in the statistical analysis in the methods section.*

**Author Response: **Thank you for your valuable comment. The relationship between individual characteristics—whether time-invariant or time-varying—and their transition rates is often a key focus in multi-state models. Explanatory variables for a specific transition intensity can be investigated by modeling the intensity as a function of these variables. Marshall and Jones described a form of proportional hazards model in which the elements of the transition intensity matrix** *q*_*rs*_​ of interest can be expressed as:



 The updated *Q* matrix is then used to compute the likelihood. When covariates *Z(t)* are time-dependent, the likelihood contributions of the form P _*rs*_ *(t* *-* *u)* are replaced by:



 This requires that the value of the covariate is known at each observation time *u*. In some cases, covariates are observed at different times than the main response, such as during recurrent disease events or through other biological markers. In such cases, covariates may be treated as step functions, remaining constant between their observation times. If the primary response (the state of the Markov process) is not observed when the covariate changes, it may be treated as a "censored" state.

 The *msm* package allows for the incorporation of both individual-specific and time-dependent covariates into transition intensities. To calculate the transition probabilities *P* *(* *t* *)*, upon which the likelihood depends, it is assumed that time-dependent covariates are constant within specific intervals. Models with transition intensities that vary over time are classified as time-inhomogeneous models.

 Marshall and Jones also described likelihood ratio tests and Wald tests for covariate selection and hypothesis testing. For instance, these tests can determine whether the effect of a covariate is consistent across all forward transitions in a disease progression model or if the effect on backward transitions is equal to the negative effect on forward transitions.

 Finally, covariates can be included in the model to examine their impact on transition rates, enabling the construction of an intensity matrix that depends on covariates. This approach is based on the assumption that covariates remain constant between the observation times of the Markov process.

 This has now been clarified in the statistical analysis section.

 References:

- Marshall G, Jones RH. Multi-state models and diabetic retinopathy. Stat Med. 1995 Sep;14(18):1975–83.
- Jackson C. Multi-State Models for Panel Data: The msm Package for R. J Stat Softw. 2011 Jan 4;38(8):1–28.

**Minor comments:**

**Comment 1:**

*In the definition of bacteremia, it would be more accurate to state that primary bloodstream infection (BSI) and catheter related BSI (CLABSI) according to the CDC criteria were included. As in CLABSI a clear source of infection is identified (catheter) and in the current phrasing is contradictory: “Infection was defined as the diagnosis of bacteremia according to the criteria given by the Center for Disease Control and Prevention (CDC), as the presence of fever, chills or hypotension with bacteria identified in the blood and not related to an infection at any other site. Central-line associated bloodstream infections (CLABSI) were also considered in the analysis”.*

**Author Response: **We fully agree with your observation. This has now been clarified in the methods section to ensure that the definition of bacteremia is more precise.

**Comment 2:**

*The last sentence in the conclusions is not supported by the study findings: ”Finally, the use of modeling tools to address this type of problem allows the continuation and improvement of active epidemiological surveillance, making it possible to identify, design and implement evidence-based strategies aimed at preventing colonization and infection in these patients.” The authors could argue instead on the utility of modelling studies to evaluate decolonization or other infection control interventions in this patient population to inform future real-world epidemiological and interventional studies.*

**Author Response: **Thank you very much for your suggestion. We agree with you, so the final part of the conclusion was modified according to your comment.

# Reviewer report: Reviewer response for version 1

**DOI:** 10.5256/f1000research.166587.r316810

**Published:** 2024-09-19

**Authors:** Stephan Harbarth, Nasreen Kheir

**Affiliations:** 1 Geneva University Hospitals, Geneva, Switzerland

Thank you for the opportunity to review the interesting study by Montoya-Urrego *et. al. *The study addresses a relevant research question on the dynamics of *Staphylococcus aureus* colonization and bloodstream infection among hemodialysis patients in a Colombian healthcare setting. The investigators used transition intensities to quantify the rate at which patients move between non-colonization, colonization and infection states. They derive transition probability matrices and intensities from patient observations, which is the mainstay of multi-state modelling.

 The study has important findings for clinicians taking care of hemodialysis patients: first *,* there is a higher probability for transition from a non-colonized to a colonized state, which highlights the challenge in *S. aureus* prevention. Second, it identifies previous infections, hospitalization, and antibiotic use as significant risk factors for transition to a colonized state, in accordance with existing literature. Last, it highlights the importance of active *S. aureus* surveillance in hemodialysis patients. All these findings have implications on infection prevention and control practice and can inform future interventions targeting this patient group, beyond conventional decolonization strategies.

 After reading the manuscript we found that while the study rationale and objectives are clearly described, there are a few important issues that need to be better addressed in the sections about methodology, results, and discussion. Furthermore, a separate Limitations section should be added to the manuscript.

**Major comments**

1. Applying Markov models to study the dynamics of colonization and infection by *S. aureus* is an interesting approach, yet there is an important limitation to this approach that should be acknowledged. For the case of *S. aureus* colonization among hemodialysis patients, the probability of colonization depends also on the number of patients colonized in the same unit, namely ‘colonization pressure’, as well as on other factors such as hospital environment and thus, it is not at all random. This cannot be accounted in such a model for and should be discussed as an important limitation of the chosen modelling approach.
2. The model’s assumption of constant transition rates over time is problematic from a clinical perspective. Acquisition probability might change according to various factors, such as hospitalization, administration of antibiotics, use of invasive instrumentation etc. As such, the clinical relevance of these findings is questionable. This is another limitation that should be discussed in the manuscript.
3. The methods section lacks important information that is critical for interpreting the study findings. The authors cite their previous publication (Vanegas et al 2021) that served as a basis for this secondary data analysis; however, essential information should be described in the current manuscript as well, for example the complete lack of decolonization measures for patients undergoing chronic hemodialysis in the studied hemodialysis clinic.
4. Selection bias might be introduced by exclusion of patients with baseline measurement only (15% of all patients). The missing measurements could be not missing at random, as hemodialysis patients have increased mortality rates and can have renal transplantation, and the follow-up is not expected to be complete. These two events are competing events that should be accounted for in the model if such data exists, otherwise they should be further discussed in the limitations section.
5. In line with the previous comment, specific information on the status of colonization and infection for the patients at the different time points assessed should be presented in a supplement. This information is crucial to assess the internal validity of the model and should be disclosed to the reader.
6. The authors should consider providing an anonymized dataset (or a synthetic data set) and their analysis code for transparency, reproducibility, and educational purposes.
7. The definition used for bacteremia state may be problematic, as it includes only a subgroup of patients with primary bloodstream infection, excluding central-line associated bloodstream infections (CLABSI). The latter constitute a substantial part of *S. aureus* bloodstream infections in this patient population. This should be addressed, and better clarified in case CLABSIs were indeed included.
8. The sampling strategy for *S. aureus* colonization is not comprehensive, as it includes only nostrils and catheter insertion site. The colonization prevalence might have been different if additional sites were screened, see also: McKinnell JA et al, Infect Control Hosp Epidemiol. 2013. As such, the sensitivity of the screening approach in the primary study should be also discussed as possible source of bias.
9. There is no mention of the distinction between MRSA and MSSA in this analysis (presented in the index publication in 2021). Analysis by MRSA/MSSA might have not been possible due to a limited number of patients with MRSA included in the original dataset, yet this should be discussed in the current study as well.

**Minor comments:**

1. The external validity of findings is limited, since this study relies on a small patient sample in a single centre hemodialysis unit of patients receiving hemodialysis through a venous catheter, with three *S.aureus* sampling events. This should be discussed as a limitation.
2. As patients are included at an arbitrary timepoint during the hemodialysis treatment, it is unclear how these transition intensities behave during the time course of hemodialysis. This also should be discussed and possible implications on the study findings should be addressed.
3. The statistical methods could benefit from more detailed explanation including the theoretical relations between P,Q, qrs, with formula and which software was used, and what packages with their respective versions.
4. The abstract needs to be revised some phrases such as ‘some variables’ and ‘high complexity hospital’ can be substituted with more precise terms and data.

**Optional suggestion to extend the current analysis:**

 The authors could consider an option of adding a complementary simulation analysis to enrich the current study. For example, a sensitivity analysis of the Markov model and/or a scenario-based analysis reflecting on specific infection prevention interventions. Possible suggestions:

- Simulation with Non-Fixed Time Intervals: a time-varying/non-homogeneous Markov model can be used to simulate a scenario where transition rates can vary over time. This would better capture periods when the risk of infection or colonization is higher (e.g., hospitalization or administration of antibiotic treatments). The results obtained can be then compared to the homogeneous model to analyze the impact of varying transition rates on the duration of colonization or infection periods.
- Simulation of the effect of preventive intervention strategies on colonization. This simulation can be used to evaluate different preventive strategies, such as improved hygiene measures or changes in care protocols (e.g., systematic disinfection of catheters/ decolonization of *S. aureus *carriers). This analysis could test the impact of such interventions on reducing transitions from non-colonization to colonization accounting for different levels of adherence to these measures and observe their effect on reducing transitions.

**Structured feedback:**

- **Is the work clearly and accurately presented and does it cite the current literature?**
  Partially, the literature review is adequate, yet additional information is needed in the methods, results and a limitations section in the discussion to improve the readability and interpretability of this manuscript.
- **Is the study design appropriate and is the work technically sound?**

Yes, the data is sparse, with few measurement points for small sample, but overall, this manuscript has a nice implementation of this modelling approach.

- **Are sufficient details of methods and analysis provided to allow replication by others?**

No, additional information is needed, and the analysis code should be shared in supplement or through an adequate repository (GitHub ,Zenodo, etc) please see specific comments above.

- **If applicable, is the statistical analysis and its interpretation appropriate?**

Yes.

- **Are all the source data underlying the results available to ensure full reproducibility?**

Yes, However, we still recommend that the number of patients with available data in each of the 3 measurement points to be reported in an aggregate manner in the manuscript.

**Are the conclusions drawn adequately supported by the results?**

 Yes.

Is the work clearly and accurately presented and does it cite the current literature?

Partly

If applicable, is the statistical analysis and its interpretation appropriate?

Yes

Are all the source data underlying the results available to ensure full reproducibility?

Yes

Is the study design appropriate and is the work technically sound?

Yes

Are the conclusions drawn adequately supported by the results?

Yes

Are sufficient details of methods and analysis provided to allow replication by others?

No

Reviewer Expertise:

Healthcare epidemiology, infection control, MSSA/MRSA

We confirm that we have read this submission and believe that we have an appropriate level of expertise to confirm that it is of an acceptable scientific standard, however we have significant reservations, as outlined above.

# Response

**Published:** 2024-10-28

**Authors:** Daniela Montoya Urrego

**Affiliations:** Escuela de Microbiología, Universidad de Antioquia, Medellín, Antioquia, Colombia

Dear reviewers,

 Thank you for your valuable feedback on our submission. We have carefully revised the manuscript and addressed all of your comments. We believe these changes have substantially strengthened the paper, and we hope it now meets the criteria for publication.

**Major comments:**

**Comment 1:**

*Applying Markov models to study the dynamics of colonization and infection by S. aureus is an interesting approach, yet there is an important limitation to this approach that should be acknowledged. For the case of S. aureus colonization among hemodialysis patients, the probability of colonization depends also on the number of patients colonized in the same unit, namely ‘colonization pressure’, as well as on other factors such as hospital environment and thus, it is not at all random. This cannot be accounted in such a model for and should be discussed as an important limitation of the chosen modelling approach.*

**Response:**

 We fully agree with your observation, so it was added to the discussion within the limitations of our study. We appreciate that you have raised this very relevant point.

**Comment 2:**

*The model’s assumption of constant transition rates over time is problematic from a clinical perspective. Acquisition probability might change according to various factors, such as hospitalization, administration of antibiotics, use of invasive instrumentation etc. As such, the clinical relevance of these findings is questionable. This is another limitation that should be discussed in the manuscript.*

**Response:**

 Thank you for your valuable comment. Although Table 2 analyses the influence of different covariates on transition risks, for example antibiotic use, previous hospitalizations, history of infections and presence of comorbidities, we recognize that assuming constant transition rates over time may overlook important clinical factors. We have addressed this limitation in the discussion section of the manuscript.

**Comment 3:**

*The methods section lacks important information that is critical for interpreting the study findings. The authors cite their previous publication (Vanegas et al 2021) that served as a basis for this secondary data analysis; however, essential information should be described in the current manuscript as well, for example the complete lack of decolonization measures for patients undergoing chronic hemodialysis in the studied hemodialysis clinic.*

**Response:**

 We agree with the observation, thank you for your comment. Our previous publication (Vanegas et al., 2021) is cited because, as the reviewers point out, it forms the foundation for the analysis in this study. However, as suggested, we complete the methods section in more detail with essential information such as the absence of decolonization protocols.

**Comment 4:**

*Selection bias might be introduced by exclusion of patients with baseline measurement only (15% of all patients). The missing measurements could be not missing at random, as hemodialysis patients have increased mortality rates and can have renal transplantation, and the follow-up is not expected to be complete. These two events are competing events that should be accounted for in the model if such data exists, otherwise they should be further discussed in the limitations section.*

**Response:**

 Thank you for your observation. We have added the reasons for patients having only one measurement in the results section. Additionally, we have included these events as a limitation in the discussion section, acknowledging that mortality and renal transplantation are competing events. In future studies, we will consider performing different types of risk analysis to account for such competing events. Thank you again for your value comment.

**Comment 5 and 6:**

*In line with the previous comment, specific information on the status of colonization and infection for the patients at the different time points assessed should be presented in a supplement. This information is crucial to assess the internal validity of the model and should be disclosed to the reader.*

*The authors should consider providing an anonymized dataset (or a synthetic data set) and their analysis code for transparency, reproducibility, and educational purposes.*

**Response:**

 A synthetic data set was added with the information requested by the reviewers (Extended Data).

**Comment 7:**

*The definition used for bacteremia state may be problematic, as it includes only a subgroup of patients with primary bloodstream infection, excluding central-line associated bloodstream infections (CLABSI). The latter constitute a substantial part of S. aureus bloodstream infections in this patient population. This should be addressed, and better clarified in case CLABSIs were indeed included.*

**Response:**

 Thank you for your observation. We confirm that central-line associated bloodstream infections (CLABSI) were indeed included in our analysis. This has now been clarified in the methodology section to ensure that the definition of bacteremia is more precise.

**Comment 8:**

*The sampling strategy for S. aureus colonization is not comprehensive, as it includes only nostrils and catheter insertion site. The colonization prevalence might have been different if additional sites were screened, see also: McKinnell JA et al, Infect Control Hosp Epidemiol. 2013. As such, the sensitivity of the screening approach in the primary study should be also discussed as possible source of bias.*

**Response:**

 We recognize that *S. aureus* colonization can occur in multiple body sites, but the nasal region is the most common and is considered a strong indicator of overall colonization. Studies show that nasal colonization correlates highly with colonization at other body sites, so focusing on the nostrils offers high sensitivity for detecting *S. aureus* colonization. However, we will take your valuable suggestion into account in future studies to improve the accuracy of detection, and we included this limitation in the discussion section. Thank you for your observation.

***References:***

- Sakr A, Brégeon F, Mège J-L, Rolain J-M, Blin O. *Staphylococcus aureus* Nasal Colonization: An Update on Mechanisms, Epidemiology,  Risk Factors, and Subsequent Infections. Front Microbiol. 2018;9:2419
- A. ML, M. CJ, Pauline C, Gail M, Dan M. Methicillin-Resistant *Staphylococcus aureus* Colonization at Different Body Sites: a Prospective, Quantitative Analysis . J Clin Microbiol. 2011 Mar 1;49(3):1119–21

**Comment 9:**

*There is no mention of the distinction between MRSA and MSSA in this analysis (presented in the index publication in 2021). Analysis by MRSA/MSSA might have not been possible due to a limited number of patients with MRSA included in the original dataset, yet this should be discussed in the current study as well.*

**Response:**

 As noted by the reviewers, a separate analysis of the behavior of methicillin-resistant *Staphylococcus aureus* (MRSA) and methicillin-susceptible *Staphylococcus aureus* (MSSA) was not possible due to the limited number of MRSA-colonized patients in the dataset. This point was added to the discussion section as a limitation.

**Minor comments:**

**Comment 1:**

*The external validity of findings is limited, since this study relies on a small patient sample in a single center hemodialysis unit of patients receiving hemodialysis through a venous catheter, with three  S. aureus sampling events. This should be discussed as a limitation.*

**Response:**

 Thanks for the comment, it has been discussed as a limitation in the manuscript. However, the institution where the research was conducted is one of the largest in the city and serves a diverse population from various areas, which increases external validity.

**Comment 2:**

*As patients are included at an arbitrary timepoint during the hemodialysis treatment, it is unclear how these transition intensities behave during the time course of hemodialysis. This also should be discussed and possible implications on the study findings should be addressed.*

**Response:**

 The hemodialysis time of each patient was not taken into account in this study, so it is discussed within the limitations. Thank you for the observation.

**Comment 3:**

*The statistical methods could benefit from more detailed explanation including the theoretical relations between P,Q, qrs, with formula and which software was used, and what packages with their respective versions.*

**Response:**

 The explanation of the statistical analysis has been improved in the methods section, including information on the software used. Thank you for your feedback.

**Comment 4:**

*The abstract needs to be revised some phrases such as ‘some variables’ and ‘high complexity hospital’ can be substituted with more precise terms and data.*

**Response:**

 The Abstract was adjusted according to your recommendations.

**Optional suggestion to extend the current analysis:**

*The authors could consider an option of adding a complementary simulation analysis to enrich the current study. For example, a sensitivity analysis of the Markov model and/or a scenario-based analysis reflecting on specific infection prevention interventions. Possible suggestions:*

- *Simulation with Non-Fixed Time Intervals: a time-varying/non-homogeneous Markov model can be used to simulate a scenario where transition rates can vary over time. This would better capture periods when the risk of infection or colonization is higher (e.g., hospitalization or administration of antibiotic treatments). The results obtained can be then compared to the homogeneous model to analyze the impact of varying transition rates on the duration of colonization or infection periods.*
- *Simulation of the effect of preventive intervention strategies on colonization. This simulation can be used to evaluate different preventive strategies, such as improved hygiene measures or changes in care protocols (e.g., systematic disinfection of catheters/ decolonization of S. aureus carriers). This analysis could test the impact of such interventions on reducing transitions from non-colonization to colonization accounting for different levels of adherence to these measures and observe their effect on reducing transitions.*

**Response:**

 Thank you for your valuable suggestions on possible complementary analyses. We agree that incorporating additional simulations would provide a clearer understanding of the dynamics of the model. In particular, it would be interesting to explore time-varying transition rates and assess the impact of infection prevention interventions. We assure you that these insights will be taken into account for future extensions of this research. For the current study, we aimed to focus on providing an initial exploration of the transitions between non-colonization, colonization, and infection using a simpler Markov model.

 Your recommendations will certainly guide us in refining and enriching our analysis in subsequent studies.

# Reviewer report: Reviewer response for version 1

**DOI:** 10.5256/f1000research.166587.r313718

**Published:** 2024-09-05

**Authors:** Thanaa Rasheed Abdulrahman

**Affiliations:** 1 Department of Microbiology, University of Al-Nahrain, Baghdad, Iraq

Dear Editor

 The answer below for both questions.

 Are sufficient details of methods and analysis provided to allow replication by others? Are all the source data underlying the results available to ensure full reproducibility?

 The author followed the Markov model in conducting the research in terms of the transition between colonization, non-colonization, and infection, but he did not explain the details of the test, how the period was calculated, and how long each transition was. The details of the work must be added to make it a reliable reference research.

 Also, the author could have followed up on the period or generation time of bacteria between the transition from non-colonized to colonized or bacteremia, and whether it can contribute to stopping the transition or introducing a treatment to stop colonization.

Is the work clearly and accurately presented and does it cite the current literature?

Yes

If applicable, is the statistical analysis and its interpretation appropriate?

Yes

Are all the source data underlying the results available to ensure full reproducibility?

Partly

Is the study design appropriate and is the work technically sound?

Yes

Are the conclusions drawn adequately supported by the results?

Yes

Are sufficient details of methods and analysis provided to allow replication by others?

Partly

Reviewer Expertise:

Microbiology, medical, clinical and diagnostic bacteriology, moleculer microbiology, immunology, parasitology

I confirm that I have read this submission and believe that I have an appropriate level of expertise to confirm that it is of an acceptable scientific standard, however I have significant reservations, as outlined above.

# Response

**Published:** 2024-10-28

**Authors:** Daniela Montoya Urrego

**Affiliations:** Escuela de Microbiología, Universidad de Antioquia, Medellín, Antioquia, Colombia

Dear reviewer,

 Thank you for guidance reviewing our submission. The manuscript has been revised and your comments have been directly addressed. We believe that these revisions lead to a significantly improved manuscript and we hope it is now acceptable for publication.

**Comment 1:**

*The author followed the Markov model in conducting the research in terms of the transition between colonization, non-colonization, and infection, but he did not explain the details of the test, how the period was calculated, and how long each transition was. The details of the work must be added to make it a reliable reference research.*

***Response:***

 The colonization screening process are detailed in the Methods section, likewise the screening periods established in the methodology were based on a previous study conducted by our research group, also considering that colonization can behave intermittently over time, appearing or disappearing at different moments. Several studies have shown that hemodialysis patients can transition between non-colonization and colonization in short periods of time, ranging from 2 weeks to 2 months after starting dialysis therapy, especially in those with catheters.

**References:**

- Scheuch M, Freiin von Rheinbaben S, Kabisch A, et al.: *Staphylococcus aureus* colonization in hemodialysis patients: a prospective 25 months observational study. *BMC Nephrol.* 2019;20(1):153. 31060511 10.1186/s12882-019-1332-z PMC6503363
- Grothe C, Taminato M, Belasco A, Sesso R, Barbosa D. Screening and treatment for *Staphylococcus aureus* in patients undergoing hemodialysis: a systematic review and meta-analysis. BMC Nephrol. 2014;15(1):202
- Agrawal V, Valson AT, Bakthavatchalam YD, Kakde S, Mohapatra A, David VG, et al. Skin Colonizers and Catheter Associated Blood Stream Infections in Incident  Indian Dialysis Patients. Indian J Nephrol. 2022;32(1):34–41

For this reason, repeated measurements were taken over time to capture these changes in patient status. While we acknowledge that conducting more frequent measurements would have been ideal, it was not possible in this study, because the budget available for the research was only sufficient for three observations over time. However, we appreciate the comment and will take it into account for future research.

 Regarding the duration of each transition, this was calculated using the transition matrix Q, in which temporal homogeneity is assumed in the transitions between states. In this study, we used a continuous-time Markov model to analyze transitions between non-colonization, colonization, and infection states. Markov models are particularly well-suited for this type of research because they allow us to model the dynamics of transitions over time based solely on the patient’s current state without considering previous states, this is a Markovian property of homogeneity. This approach assumes that once the patient enters a given state, the probability of moving to a new state depends only on that current state and not on any  prior states. This allows us to calculate the probabilities of changing from one state to another based on the patient's current state, maintaining the constancy of the transition probabilities over time. The methods section was enhanced in the manuscript to better explain this part.

**Comment 2:**

*Also, the author could have followed up on the period or generation time of bacteria between the transition from non-colonized to colonized or bacteremia, and whether it can contribute to stopping the transition or introducing a treatment to stop colonization.*

**Response:**

 Given the intermittent nature of colonization, measurements were taken at short intervals to capture and identify all potential transitions, including the onset of bacterial colonization. For example, we observed cases where patients initially showed a non-colonization status at baseline but transitioned to colonization at subsequent measurements. Importantly, all patients started follow-up without bacteremia, and follow-up continued until the first episode of bacteremia, so the occurrence of bacteremia did not influence data on transitions between non-colonization and colonization. Furthermore, it is worth mentioning that the renal unit did not have a decolonization protocol in place, meaning that patients were not treated to control colonization. Treatment was only started when an infection was present. However, antibiotic consumption was included in the state-to-state transition analysis in order to assess the influence of this covariate on transition risk (Table 2).
